# Cutaneous adverse events in patients treated with PD-1/PD-L1 checkpoint inhibitors and their association with survival: a systematic review and meta-analysis

**DOI:** 10.1038/s41598-022-24286-3

**Published:** 2022-11-21

**Authors:** Fangmin Zhao, Junjing Zhu, Rui Yu, Tianyu Shao, Shuyi Chen, Gaochenxi Zhang, Qijin Shu

**Affiliations:** 1 The First School of Clinical Medicine, Zhejiang Chinese Medical University, Hangzhou, 310053 Zhejiang China; 2 Department of Oncology, Jiaxing TCM Hospital Affiliated to Zhejiang Chinese Medical University (Jiaxing Hospital of Traditional Chinese Medicine), Jiaxing, 314033 Zhejiang China; 3 Department of Oncology, The First Affiliated Hospital of Zhejiang Chinese Medical University (Zhejiang Provincial Hospital of Traditional Chinese Medicine), Hangzhou, 310006 Zhejiang China; 4 Cancer Institute of Integrative Medicine, Zhejiang Chinese Medical University, Hangzhou, 310053 Zhejiang China

**Keywords:** Tumour immunology, Outcomes research, Cancer immunotherapy

## Abstract

Immune-related cutaneous adverse events (irCAEs) in patients treated with programmed cell death-1/programmed death-ligand 1 (PD-1/PD-L1) checkpoint inhibitors may be associated with better clinical outcomes. However, the extent to which these results can be extrapolated to all tumour types remains unclear. Herein, we conducted a meta-analysis of patients with cancer receiving anti-PD-1/PD-L1 immunotherapy, to determine the cumulative incidence of irCAEs and their association with survival. We systematically searched six databases (PubMed, Embase, Cochrane, CNKI, CSPD, and CQVIP database) for all cohort studies reporting the relationship between irCAEs and patient survival from the time of database construction to 1 November, 2020. The primary outcomes were objective response rate (ORR), progression-free survival (PFS), and overall survival (OS), with complete remission (CR), partial remission (PR), stable disease (SD), and progressive disease (PD) as secondary outcomes. Patients with irCAEs exhibited higher ORR, and were more likely to report CR and PR and less likely to develop PD than those who did not experience irCAEs. Moreover, the occurrence of irCAEs was significantly associated with both favourable PFS and OS. Therefore, patients with irCAEs have better survival benefit and a significantly lower risk of tumour progression or death. Hence, the occurrence of irCAEs may be a useful marker for predicting the clinical efficacy of anti-PD-1/PD-L1 immunotherapy.

## Introduction

Immune checkpoint inhibitors (ICIs) have been introduced as breakthroughs in cancer treatment. Programmed cell death-1/programmed death-ligand 1 (PD-1/PD-L1) checkpoint inhibitors are the most commonly used ICIs. They can inhibit the activation of the PD-1/PD-L1 pathway by binding to PD-1 or PD-L1 and then activate cytotoxic T cells and other immune cells to kill tumour cells^1^. So far, the FDA has approved seven PD-1/PD-L1 checkpoint inhibitors (nivolumab, pembrolizumab, cemiplimab, dostarlimab, atezolizumab, avelumab, and durvalumab) for non-small cell lung cancer (NSCLC), bladder cancer, oesophageal cancer, melanoma, and other cancer types^2,3^.

Patients receiving PD-1/PD-L1 checkpoint inhibitor therapy have a better survival time and an unprecedentedly higher cure rate than those treated with conventional chemotherapy^4–7^. However, immune-related adverse events (irAEs) secondary to PD-1/PD-L1 checkpoint inhibitor treatment are not uncommon, and dermatological toxicities are one of the most common irAEs among all the organ systems^8–10^ and constitute a major challenge in clinical decision making. They are observed in more than one-third of the treated patients^11^, seriously affecting their quality of life, mental health, and survival^12^. Clinically, immune-related cutaneous toxic effects mainly include skin rashes, pruritus, and vitiligo. In severe cases, such as Stevens-Johnson syndrome (SJS) or toxic epidermal necrolysis (TEN), drug rash or reactions resulting in eosinophilia and systemic symptoms (DRESS) can be life-threatening^13,14^. Management guidelines such as the Multinational Association of Supportive Care in Cancer (MASCC), National Comprehensive Cancer Network (NCCN), and the European Society for Medical Oncology (ESMO)^15,16^ all recommend suspension or permanent discontinuation of immune checkpoint inhibitors when severe irCAEs occur. Despite the hazards of immune-related cutaneous adverse events (irCAEs), some studies suggest that patients with irCAEs may benefit more from treatment with PD-1/PD-L1 checkpoint inhibitors^17,18^; however, the extent to which these results can be extrapolated to all tumour types remains unclear. Therefore, we performed a systematic review and meta-analysis of published studies to quantify the association between irCAEs and survival in patients treated with PD-1/PD-L1 checkpoint inhibitors. This review provides insights for a better understanding of irCAEs to facilitate clinical application and risk and overall management of PD-1/PD-L1 checkpoint inhibitors in patients.

## Results

### Characteristics of included studies

**Figure 1:**
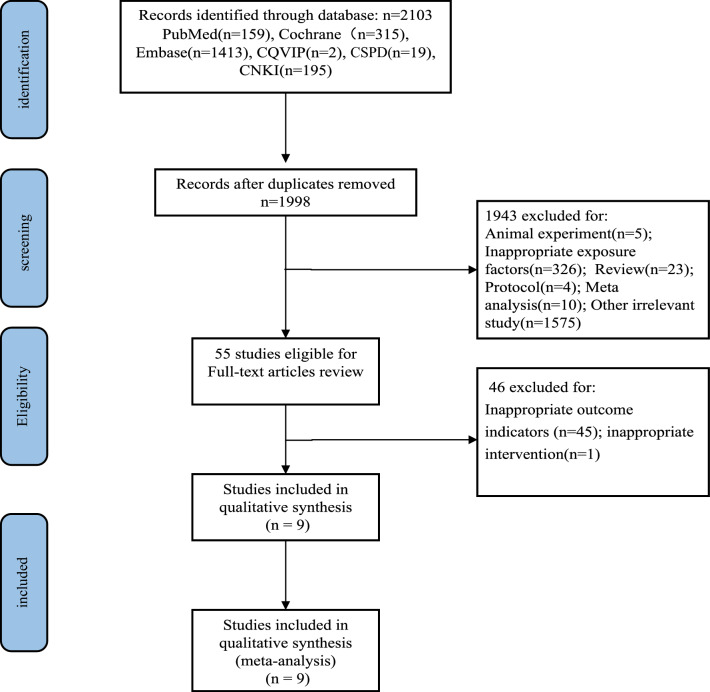
Study selection process.

A total of 2103 studies were initially selected. After removing duplicates and screening the titles and abstracts, 55 publications were identified; of these, 46 were excluded because they did not meet the inclusion criteria. In total, nine studies (with data from 2105 participants) were included in our final analysis (Fig. 1). The general characteristics of the nine included studies are summarised in Supplementary Table S1. All studies were published between 2016 and 2020 (four were published in 2020 and three were published in 2019)^1,8–10,18–22^. There were two studies from the United States^10,20^, three from Japan^8,18,19^, one each from France^21^, Australia^22^, and Korea^1^, and one each from Europe, North America, and the Asia–Pacific region^9^. Seven^1,8–10,18–20^ were retrospective, while two^21,22^ had a prospective cohort design. Five studies^10,19–22^ included patients with only melanoma, two studies^8,18^ included patients with only NSCLC, one study^9^ included patients with only bladder cancer, and one study^1^ involved patients with various cancers (renal cell carcinoma, lung cancer, gastric cancer, melanoma, colorectal cancer, bladder cancer, hepatocellular carcinoma, oesophageal cancer, oropharyngeal cancer, prostate cancer, and tonsillar cancer). The tumour stage of all patients was advanced (stage III/IV) except for one patient with stage IIA NSCLC in Akano’s study^8^, and the patients’ tumour stages were not reported in Lee’s study^1^. In eight studies^1,8,10,18–22^, patients were treated with PD-1 checkpoint inhibitors. The participants of another study were treated with the PD-L1 checkpoint inhibitor atezolizumab^9^. A total of 368 patients received PD-1/PD-L1 checkpoint inhibitors as first-line therapy and 619 patients received PD-1/PD-L1 checkpoint inhibitors as second-line or later therapy after radiotherapy, chemotherapy, targeted therapy, and immunotherapy. Three studies^9,10,22^ (including 1118 patients) did not clearly report whether PD-1/PD-L1 checkpoint inhibitors were used as first-line therapy or as second- or later lines of therapy.

### Quality of included studies

All cohort studies were evaluated using the Newcastle–Ottawa Scale (NOS) and found to be of good quality and presented low risk of bias based on total scores higher than or equal to 7 (Supplementary Table S2). All studies were rated as representative participants from real-world or clinical trials. All but one study^9^ reported adequate outcome ascertainment.

### Occurrence of irCAEs

The clinical manifestations of irCAEs included rash (macular–popular or eczematous), pruritus, vitiligo, vitiligo-like depigmentation, psoriasis, atopic dermatitis, lichenoid reaction, erythema, dry mouth, urticaria, dry skin, alopecia, and hyperpigmentation. Seven articles^1,8–10,18,19,21^ reported that patients exhibiting irCAEs were diagnosed and graded based on the National Cancer Institute’s Common Terminology Criteria for Adverse Events (NCI-CTCAE, version 4.0). The diagnosis of irCAEs in one article^22^ was made jointly by oncologists and dermatologists based on clinical features and supported using histopathology when necessary. One article^20^ did not report their diagnostic criteria for irCAEs. The number, NCI-CTCAE grade, and types of irCAEs are summarised in Supplementary Table S3.

A total of 1217 patients were evaluated across eight studies that reported the incidence of irCAEs ^1,8,10,18–22^. From the grade or type of irCAE, the incidence of irCAEs ranged from 16.6 to 58.1%, with a cumulative incidence of 387/1217 (31.8%), and a summary estimate of the incidence of 31.2% (95% CI 21.3–41.0%, I^2^ = 93.4%, *P* < 0.001). Based on data from six studies that reported the number of cases with different grades of irCAEs^1,8,10,18,19,21^, the cumulative incidence of grades 1–2 irCAEs was 218/817 (26.7%), and the incidence of grades 3–4 irCAEs was 16/817 (2.0%). The median time of onset of irCAEs was 5.4–20.8 weeks in five studies^1,9,10,18,21^. However, the data were not merged in our analysis because of the differences in the units of median time used in these studies.

### Analysis of irCAE occurrence and tumour response

**Figure 2:**
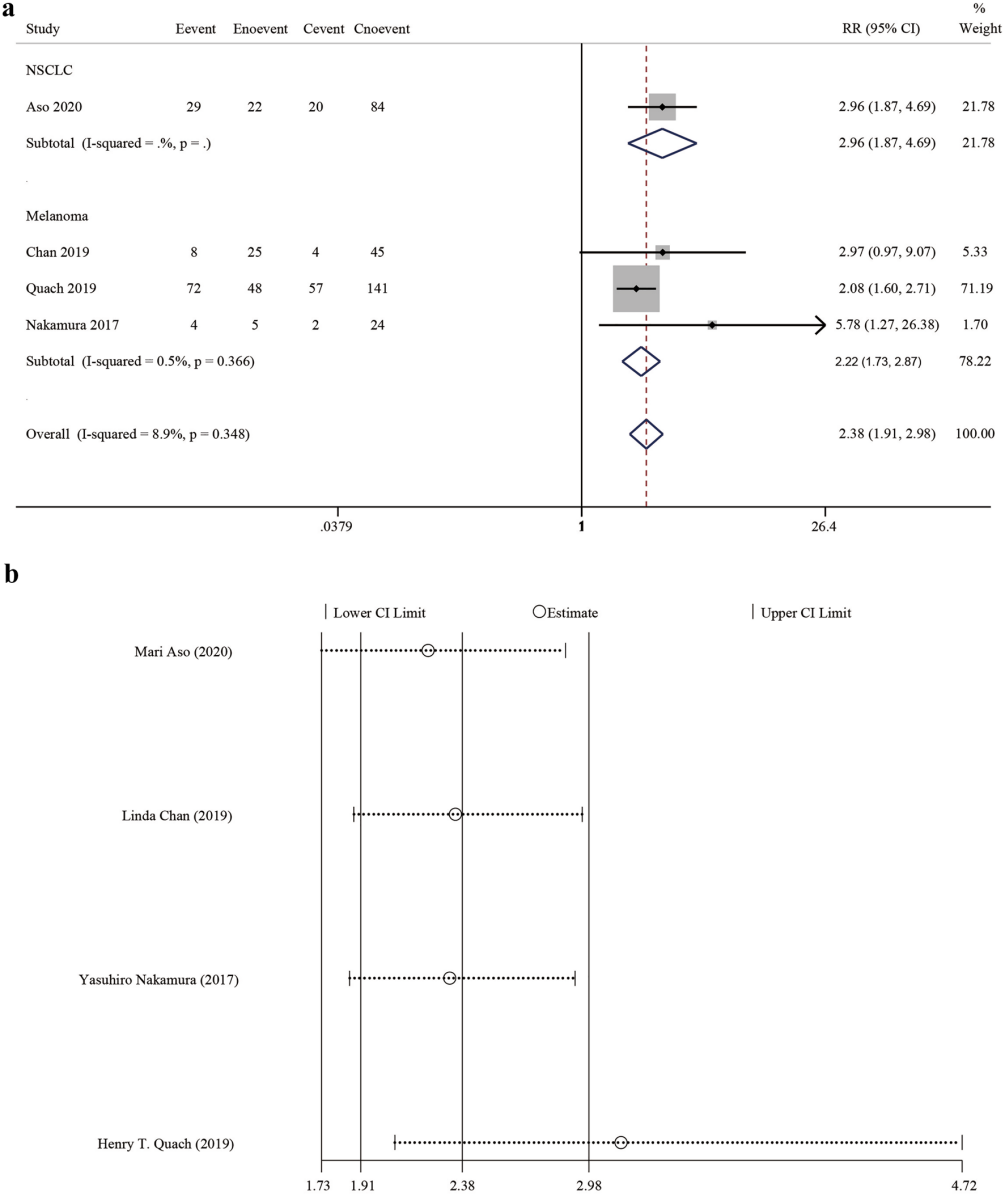
Forest plot showing the association between irCAE occurrence and ORR. (**a**) Subgroup analysis among cancer types and the ORR. (**b**) Sensitivity analysis of the ORR. The size of each square is proportional to the study’s weight. Horizontal lines indicate the 95% CIs. Diamonds indicate the pooled effect size with their corresponding 95% CIs. IrCAEs, immune-related cutaneous adverse events; ORR, objective response rate; CI, confidence interval; RR, relative risk.

Four studies^18–20,22^ reported the ORR, with a pooled estimated relative risk (RR) of 2.38 (95% CI 1.91–2.98, z = 7.68, *P* < 0.001) with no significant heterogeneity (*P* = 0.348, I^2^ = 8.9%). The pooled RR of the melanoma subgroup was 2.22 (95% CI 1.73–2.87, z = 6.18, *P* < 0.001), while that of the NSCLC subgroup was 2.96 (95% CI 1.87–4.69, z = 4.61, *P* < 0.001) (Fig. 2a). This indicated that the occurrence of irCAEs was positively correlated with the ORR.

A sensitivity analysis was conducted on four studies that reported the ORRs. The results showed that the point estimate after deleting the Quach study (2019) was outside the 95% CI of the total effect, suggesting that this study had a greater impact on the combined effect (Fig. 2b). No publication bias was found based on funnel plots, Begg’s rank correlation test (*P* = 0.308) and Egger’s linear regression test (*P* = 0.146). Evaluating the Quach 2019 study in more detail, it performed well in the cohort selection, comparability, and outcome assessment, with a high NOS score; therefore, we believe the data in that article to be reliable. The weight of that study in our meta-analysis was relatively large; thus, it was further tested using the trim-and-fill method (Supplementary Fig. F1). We found that the results did not reverse after two studies were supplemented, which indicated that the results were relatively robust; therefore, this article was not removed.

**Figure 3:**
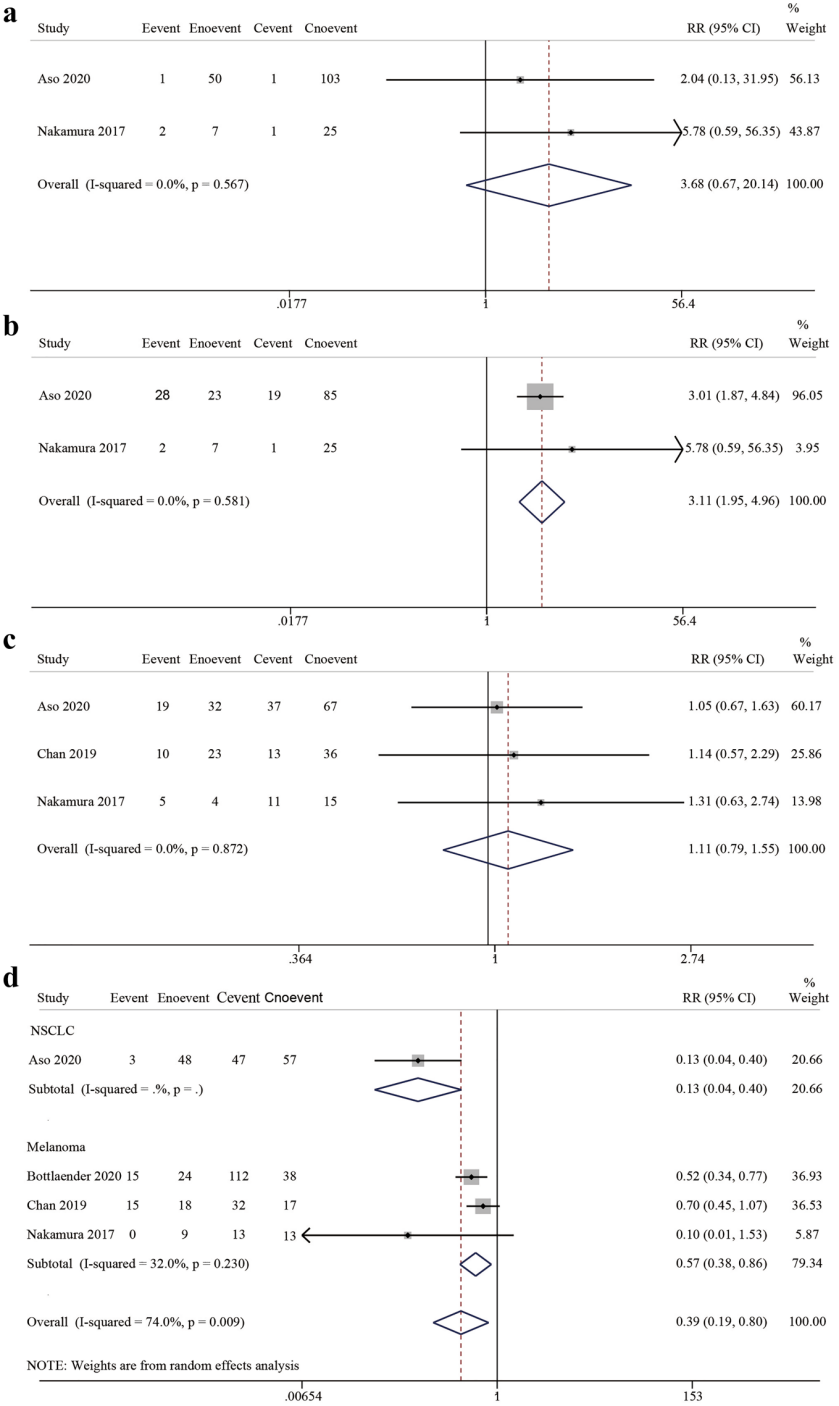
Forest plot showing the association between irCAE occurrence and CR, PR, SD, and PD. (**a**) CR, (**b**) PR, (**c**) SD, and (**d**) subgroup analysis among cancer types and PD. IrCAEs, immune-related cutaneous adverse events; CR, complete remission; PR, partial remission; SD, stable disease; PD, progressive disease.

We further evaluated the relationship between the occurrence of irCAEs and the rate of complete remission (CR), partial remission (PR), stable disease (SD), and progressive disease (PD). For the CR and PR from two studies^18,19^, the pooled estimate relative risk was 3.68 (95% CI 0.67–20.14, z = 1.50, *P* = 0.133) (Fig. 3a) and 3.11 (95% CI 1.95–4.96, z = 4.78, *P* < 0.001) (Fig. 3b), respectively, with no significant heterogeneity (*P* = 0.567, I^2^ = 0.0% and *P* = 0.581, I^2^ = 0.0%, respectively). Three studies reported SD, with a pooled estimate RR of 1.11 (95% CI 0.79–1.55, z = 0.60, *P* = 0.545) and no significant heterogeneity (*P* = 0.872, I^2^ = 0.0%) (Fig. 3c). Four studies reported that, for PD, the Q and I^2^ statistics tests showed moderate heterogeneity (*P* = 0.009, I^2^ = 74.0%) using a random-effects model analysis, with an RR of 0.39 (95% CI 0.19–0.80, z = 2.57, *P* = 0.010). We then performed subgroup analysis and found that the occurrence of irCAEs and PD in the melanoma subgroup had a direct negative correlation (RR = 0.57, 95% CI 0.38–0.86, z = 2.70, *P* = 0.007) with no significant heterogeneity (*P* = 0.230, I^2^ = 32.0%) (Fig. 3d). The NSCLC subgroup included only one study. Therefore, our subgroup analyses suggested that the cancer type was a potential source of heterogeneity between studies.

### Analysis of the association between irCAE occurrence and survival

**Figure 4:**
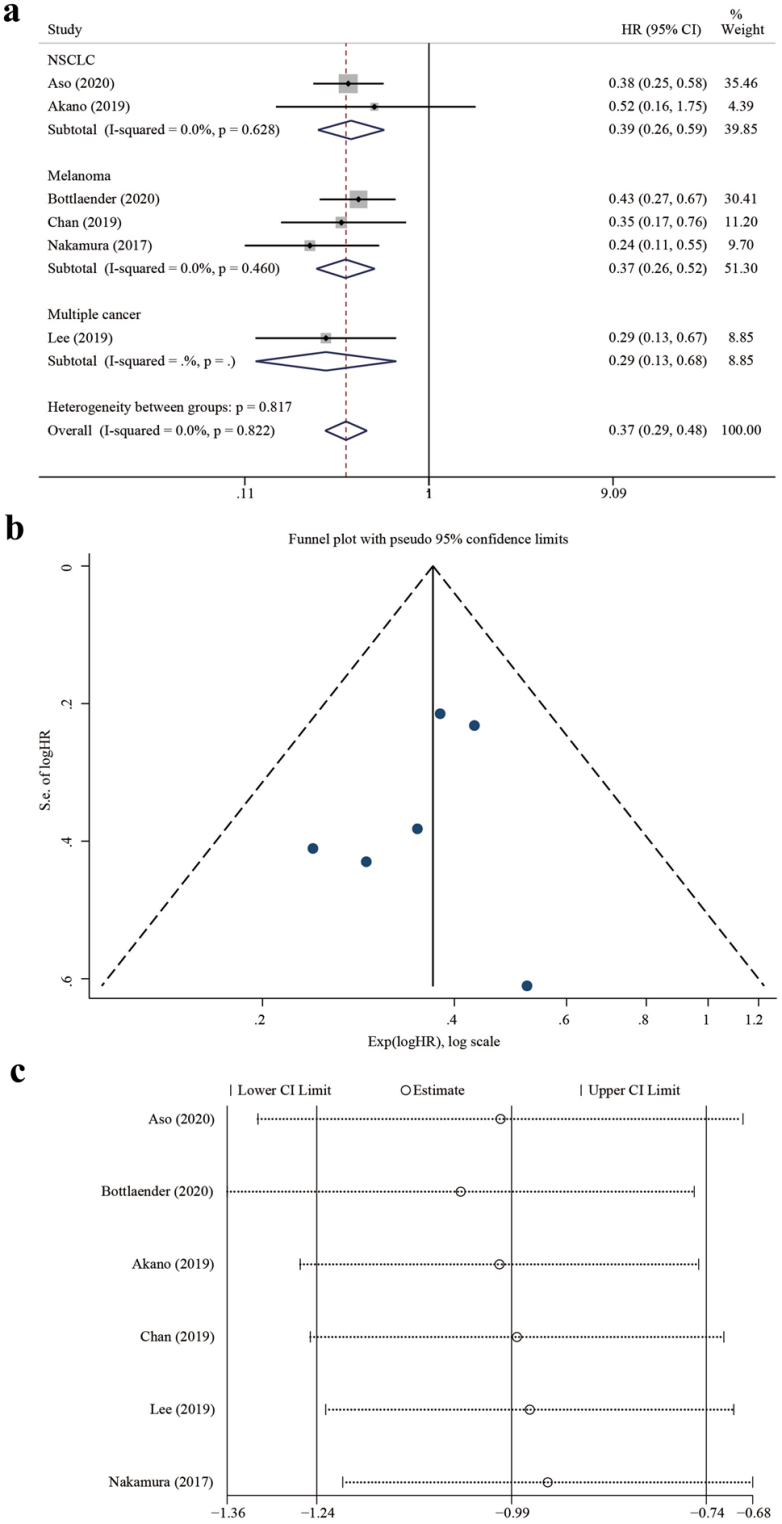
Forest plot showing the association between irCAE occurrence and PFS, publication bias analysis, and sensitivity analysis. (**a**) Subgroup analysis among cancer types and PFS. (**b**) Funnel plot of the PFS. (**c**) Sensitivity analysis of the PFS. IrCAEs, immune-related cutaneous adverse events; PFS, progression-free survival; HR, hazard ratio.

Six studies^1,8,18,19,21,22^ directly or indirectly provided the PFS with HRs and 95% CIs. The median PFS of patients who developed irCAEs ranged from 12.9–26.5 months, while it was only 3.5–3.7 months in patients without irCAEs. The pooled HR was 0.37 (95% CI 0.29–0.48, z = 7.77, *P* < 0.001) with no significant heterogeneity (*P* = 0.822, I^2^ = 0.0%). In the subgroup analyses, the HR of the NSCLC group was 0.39 (95% CI 0.26–0.59, z = 4.61, *P* < 0.001; heterogeneity test: *P* = 0.628, I^2^ = 0.0%), that of the melanoma group was 0.37 (95% CI 0.26–0.52, z = 5.60, *P* = 0.000; heterogeneity test: *P* = 0.460, I^2^ = 0.0%), and that of the multiple cancer group was 0.29 (95% CI 0.13–0.68, z = 2.87, *P* = 0.004; this subgroup only had one study). In these six studies, the patients were treated with PD-1 checkpoint inhibitors (Fig. 4a).

No publication bias was found based on the symmetrical funnel plot (Fig. 4b), Begg’s rank correlation test (*P* = 0.707), and Egger’s linear regression test (*P* = 0.49). The sensitivity analysis results showed no significant change in the combined effect of PFS after excluding any study, suggesting that the results of our study were robust (Fig. 4c).

**Figure 5:**
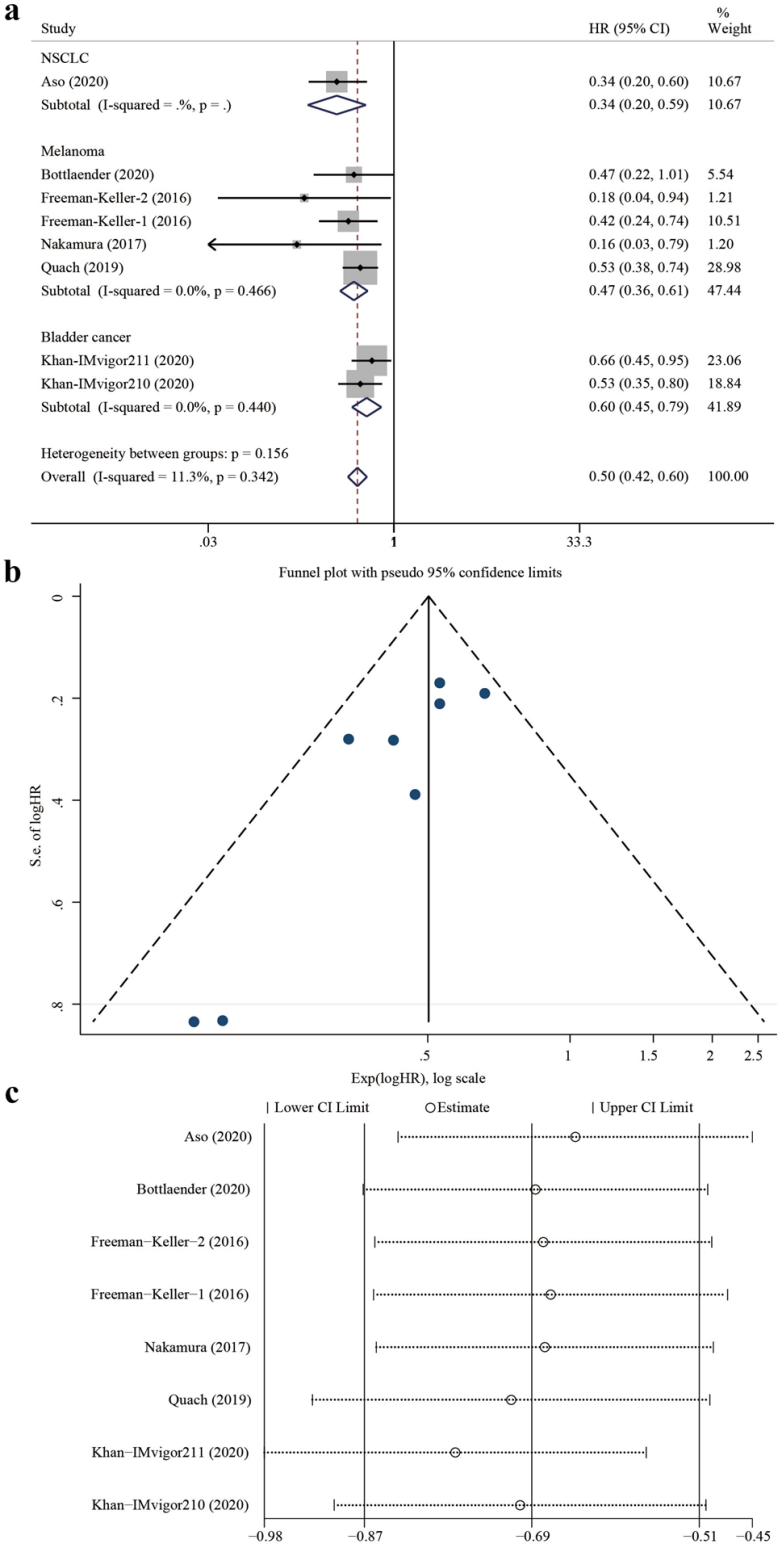
Forest plot showing the association between irCAE occurrence and OS, publication bias analysis, and sensitivity analysis. (**a**) Subgroup analysis among cancer types and OS. (**b**) Funnel plot of the OS. (**c**) Sensitivity analysis of the OS. IrCAEs, immune-related cutaneous adverse events; OS, overall survival; Freeman-Keller-1, OS data for rash; Freeman-Keller-2, OS data for vitiligo.

Six studies^9,10,18–21^ provided OS data with the HRs and 95% CIs either directly or indirectly. One of them^9^ provided two separate datasets from the clinical trial (IMvigor211 and IMvigor210), while another^10^ provided data on rash and vitiligo. The median OS of patients who experienced irCAEs ranged from 56.3 months to not reached, while it was only 11.4–17.5 months in patients without irCAEs. The pooled HR was 0.50 (95% CI 0.42–0.60, z = 7.53, *P* < 0.001), with no significant heterogeneity (*P* = 0.342, I^2^ = 11.3%). In the subgroup analyses, the HR of the NSCLC group was 0.34 (95% CI: 0.20–0.59, z = 3.85, *P* < 0.001; only one study was included), that of the melanoma group was 0.47 (95% CI 0.36–0.61, z = 5.69, *P* < 0.001; heterogeneity test: *P* = 0.466, I^2^ = 0.0%), while that of the bladder cancer group was 0.60 (95% CI 0.45–0.79, z = 3.64, *P* < 0.001; heterogeneity test: *P* = 0.440, I^2^ = 0.0%) (Fig. 5a). These results suggest that the occurrence of irCAEs can significantly reduce the risk of disease progression or death in patients with cancer (the risk was less than half that in patients without irCAEs), regardless of the cancer type.

The asymmetric funnel plot (Fig. 5b) and Egger’s linear regression test (*P* = 0.016) indicated possible publication bias. However, performing sensitivity analysis and the trim-and-fill method did not change the average effect size, further suggesting that the results were not affected by publication bias (Fig. 5c).

## Discussion

This systematic review and meta-analysis pooled nine cohort studies to present a relatively comprehensive overview of the association between irCAEs and clinical outcomes in patients treated with PD-1/PD-L1 checkpoint inhibitors. We calculated the pooled incidence rate of irCAEs to be 31.2% and that grade 1–2 irCAEs were predominant. The management of irCAEs in the nine studies included the use of moisturisers, antihistamines, and topical or systemic corticosteroid therapy^1,10,18,20,21^. Although severe irCAEs (grade ≥ 3) occurred in only a few PD-1/PD-L1 checkpoint inhibitor-treated patients with tumours, they had a significant impact on patients, especially their health-related quality of life. These effects include limiting the instrumental activities of daily living (ADL) in those with grade 2 irCAEs and self-care ADL in those with grade 3 irCAEs based on the NCI-CTCAE v5.0.

Discontinuation of PD-1/PD-L1 checkpoint inhibitors due to irCAEs was rarely reported in the included studies. One study^21^ reported that 2/39 patients with irCAEs stopped anti-PD-1/PD-L1 treatment due to irCAEs. Another study^1^ reported that none of the 35 patients with irCAEs stopped treatment with PD-1/PD-L1 checkpoint inhibitors. Other studies have not reported whether treatment with PD-1/PD-L1 inhibitors have been discontinued. However, according to the prevailing management guidelines^15,16,23,24^, patients with grade 2 irCAEs need to suspend anti- PD-1/PD-L1 immunotherapy when necessary, while those with grade 3–4 irCAEs must withhold treatment immediately or even discontinue treatment permanently. Therefore, patients with intolerable grade 2 and 3–4 irCAEs require prompt identification and appropriate management to prevent potentially life-threatening outcomes or unnecessary permanent cessation of life-saving immunotherapy. Finding a safe and effective method to treat irCAEs caused by PD-1/PD-L1 checkpoint inhibitors without reducing the intensity of anti-tumour treatment should become the focus of further research.

Our study found that the occurrence of irCAEs was significantly positively correlated with ORR, and that the PFS and OS of patients with irCAEs were significantly prolonged compared to those without irCAEs. We further analysed the relationship between the occurrence of irCAEs and other therapeutic indicators, such as CR, PR, SD, and PD. We found that the rates of CR, PR, and SD in patients who experienced irCAEs showed a trend of improvement compared with those in the non-irCAEs group, in which, the PR rate was significantly increased (*P* < 0.001) and the PD rate was significantly decreased (*P* = 0.010). To address potential heterogeneity in the populations in the selected studies, we conducted heterogeneity and subgroup analyses of the above results. Moderate heterogeneity was found in the analysis of the association between the occurrence of irCAEs and PD, while subgroup analysis suggested that cancer type was a potential source of heterogeneity between studies. Therefore, the random-effects model was used for correction. Other studies included in the analysis had good homogeneity. As there are differences in tumour biology, therapeutic response, and OS across tumour types, we standardised the subgroup analysis by tumour type. The results showed that there was no significant difference among tumour types.

Additionally, in our meta-analysis, only one patient had stage IIA NSCLC^8^, 211 patients did not report their disease stage^1^, and the other 1839 patients had advanced tumours (stage III–IV). Based on the above results, we believe that the occurrence of irCAEs indicates a better tumour response and prognosis in patients with advanced tumours treated with PD-1/PD-L1 checkpoint inhibitors.

As the original individual data of patients were not obtained, further regression analysis on the occurrence of irCAEs and its relationship with the efficacy of PD-1/PD-L1 checkpoint inhibitors as first-line or subsequent-line treatment could not be conducted directly. We then carefully reviewed the nine included studies and found that four of them^8,10,18,20^ used multivariate regression analyses (logistic regression analysis or Cox regression analysis) to prove that, after controlling for confounding factors such as previous treatment, Eastern Cooperative Oncology Group Performance (ECOG) status, age, and sex, irCAEs were independently associated with better tumour response and longer survival. Three studies^1,19,21^ reported that there was no significant difference between the irCAEs and non-irCAEs groups in terms of prior treatment. Based on the original analysis included in this study, we believe that even when PD-1/PD-L1 checkpoint inhibitors were used as the third-, fourth-, or later lines of treatment, the occurrence of irCAEs still indicates better anti-tumour treatment efficacy if parameters such as ECOG status showed improvement. We will continue to contact the authors of the relevant studies to obtain original primary data and include more eligible studies for meta-analysis in the future. We will also conduct observational clinical trials on the tumour response and survival data of patients with irCAEs after treatment with PD-1/PD-L1 checkpoint inhibitors in different lines of treatment.

Recently, immunotherapy has become mainstream and shown significant efficacy in tumours with PD-1/PD-L1 immunosuppressive mechanisms, such as malignant melanoma, Hodgkin’s lymphoma, and some lung and colon cancers^25^. The latest NCCN guidelines have included immunotherapy as a first-line treatment strategy for certain tumours. For example, pembrolizumab is recommended as a first-line treatment for patients with locally advanced or metastatic NSCLC with PD-L1 positivity. Pembrolizumab or nivolumab is recommended as a first-line treatment for dMMR/MSI-H colon cancer and advanced or unresectable melanoma^26–28^. We found that the occurrence of irCAEs was strongly positively correlated with therapeutic benefit; therefore, it is necessary to study the pathophysiological mechanisms of irCAEs.

Among all types of irCAEs, the incidence of vitiligo after immunotherapy in patients with melanoma has been the most frequently reported, with consistent results. Immune-related vitiligo in patients with melanoma is associated with pigment loss due to epidermal melanocyte 's number reduction^29^. Studies have shown that normal melanocytes express the same melanosome proteins (e.g., tyrosinase, MART-1, gp100, tyrosinase-related protein-1 (TRP-1) and TRP-2, among others) as melanoma cells, which were the self-antigens inducing antitumor immune responses, while the vitiligo regions of malignant melanoma patients with immune-related vitiligo share the same infiltration of CD8^+^ T cell clones as patients with melanoma^30,31^. The PD-L1/PD-1 pathway may mediate peripheral tolerance of the body to the aforementioned self-antigens^32^. Blocking this pathway may lead to the overexpression and release of melanoma-related antigens and increase T cell activity, which will decrease peripheral immune tolerance to melanoma-related antigens^33^. Cytotoxic T cell responses against these self-antigens not only exert an anti-tumour effect but also destroy melanocytes that express normal levels of tyrosinase, TRP, and other proteins, leading to the occurrence and persistence of autoimmune vitiligo^10,19^. In murine models, vitiligo-affected hosts were also found to maintain gp100- and TRP-2-specific memory CD8^+^ T cell levels tenfold more frequently than unaffected hosts^34^. In addition, TRP-2, which is highly expressed in melanomas and melanocytes, has been shown to induce antibody responses. High anti-TRP-2 IgG titres have been found in patients with malignant melanoma, vitiligo, and active-specific immunotherapy-induced depigmentation^35^. Another study suggested that irCAEs are related to PD-1/PD-L1 checkpoint inhibitors that block PD-1 receptors and enhance the activation of B cells to dermal or epidermal antigens^15^. This suggests that the humoral immune response may be the underlying pathogenetic mechanism of vitiligo in patients with non-melanoma tumours receiving anti-PD-1/PD-L1 immunotherapy.

Few studies have been conducted on the mechanisms of immune-related rashes. Amarnath et al.^36^ and Fujiwara et al.^37^ used mouse models of acute and chronic graft-versus-host disease (GVHD) and found that regulatory T cells (Tregs) promote immunosuppression through PD-L1 upregulation leading to GVHD and that the PD-1/PD-L1 pathway inhibits Th17/Th1-mediated chronic GVHD. Typical skin changes caused by PD-1/PD-L1 checkpoint inhibitors, such as rashes, are similar to GVHD. Dulos et al.^38^ found that treating patients with advanced melanoma or prostate cancer with PD-1/PD-L1 checkpoint inhibitors can switch antigen-induced cellular reactivity to proinflammatory Th1/Th17 responses, such as enhancing the production of interferon-γ, interleukin (IL)-2, tumour necrosis factor α, IL-6 and IL-17, and decreasing the production of Th2 cytokines IL-5 and IL-13. This suggests that immune-related rashes may be related to the action of Tregs, Th1/Th17 cells, and related cytokines.

Studies on immune-related dermatitis have shown that it is a clinical symptom caused by lymphocyte infiltration and recruitment and enhanced immune activation, which suggests a strong anti-tumour response^39^. The lichenoid reaction is a typical histopathological manifestation mediated by CD4^+^ and CD8^+^ T cells^40,41^. In contrast, there is currently no specific study on the mechanism of pruritus, one of the most prevalent irCAEs caused by PD-1/PD-L1 checkpoint inhibitors. Liniker et al. suggested that anti-PD-1 therapy may stimulate PD-L1 expression in keratinocytes and allow activated cytotoxic CD8^+^ T cells to target keratinocytes, leading to keratinocyte apoptosis, resulting in SJS^42^. In addition, Goldinger et al. demonstrated the upregulation of cytotoxic mediators (such as PRF1 and GZMB) and major inflammatory chemokines (such as CXCL9, CXCL10, and CXCL11) using gene expression analysis from TEN-like lesional skin from patients treated with anti-PD-1 therapies^43^. In summary, the action of PD-1/PD-L1 checkpoint inhibitors and immune-related cells not only exert anti-tumour effects but also act on related antigens and cytokines, causing irCAEs^44^.

The limitations of our study include its observational and retrospective nature. Of the studies included in our meta-analysis, 77.8% were retrospective, all of which depend on the accuracy and integrity of the medical records. This variation may be caused by measurement bias, selection bias, confounding, and differences in effect modification^45^. Of note, the manifestations of irCAEs may be variable, and most studies are based on patients’ and researchers’ levels of awareness regarding irCAEs. Moreover, grades 1–2 irCAEs may be considered non-serious autoimmune toxicities, which was not necessary to report. In our review, only one study indicated that the determination of irCAEs occurred through skin biopsies, while two studies reported diagnoses performed by dermatologists and/or oncologists. However, it is usually unclear whether appropriate skin examinations were performed and whether the doctors could accurately diagnose the type of irCAEs of patients. Therefore, we may have underestimated the incidence of irCAEs and overestimated their impact on the efficacy of anti-tumour therapy in this meta-analysis.

In addition, almost all of the nine studies included in our review included patients with advanced-stage cancers (stage III-IV), and that PD-1/PD-L1 checkpoint inhibitors were used as first-line treatment or as second- or third-line treatments. However, the data for each line of treatment were not reported separately, so the doses and courses of treatment could not be unified. Therefore, this study cannot provide a definitive conclusion on whether there are differences in the incidence of irCAEs in each line of treatment, its correlation with the outcome of each line of treatment, or whether there is a dose-time-dependent relationship between the occurrence of irCAEs and its clinical benefits.

We also found that the occurrence of irCAEs was positively correlated to patient survival, irrespective of tumour type. However, due to the limited number of included studies, it is unclear whether there are differences in the correlation between irCAEs and survival across different tumours, between different types of irCAEs and survival, or between irCAEs and survival in patients treated with different PD-1/PD-L1 checkpoint inhibitors. At the same time, most of the included studies did not report the tumour response or survival data of patients with different grades of irCAEs; therefore, we failed to obtain the relationship between irCAE severity and efficacy. Larger scale studies would allow the examination of clinical outcomes in each line of immunotherapy for each cancer type, as different cancers may have varying clinical processes. Moreover, large studies could also match PD-1/PD-L1 checkpoint inhibitors and irCAE types. Despite these limitations, evidence-based medicine is important in the era of precision medicine; therefore, it is necessary to perform this meta-analysis.

Overall, our meta-analysis supports the clinical benefit of irCAEs in patients receiving anti-PD-1/PD-L1 immunotherapy. Compared with non-irCAE patients, patients with irCAEs had a higher ORR and a lower risk of disease progression and death. This study also supports the significance of irCAEs as clinical markers for effective anti-tumour immunity and clinical outcomes after immunotherapy in patients with various tumours. After summarising the current research results on the pathophysiological mechanism of irCAEs, we believe that various cell types, related cytokines, and antigenic proteins participate in the pathogenesis of irCAEs, including CD4^+^ and CD8^+^ T cells, Th1/Th17 cells, Tregs, B cells, CXCL9, CXCL10, CXCL11, IL-6, IL-10, tyrosinase, MART-1, gp100, TRP-1, and TRP-2, among others^46,47^. However, since the underlying pathophysiological mechanisms of irCAEs have not yet been fully elucidated, further studies are still required.

## Methods

Our systematic review and meta-analysis were based on the preferred reporting items for systematic review and meta-analysis (PRISMA) guidelines^48^ and were registered in PROSPERO (CRD42021250251).

### Search strategy

We systematically searched the PubMed, Excerpt Medica Database (Embase), Cochrane Database of Systemic Review, China National Knowledge Infrastructure (CNKI), Wanfang Database of China Science Periodical Database (CSPD), and the Chongqing VIP (CQVIP) databases from inception to 1 November 2020, without language restrictions. The search strategy was based on the PICOS principle, using a combination of the medical subject heading (MeSH) terms and entry terms ‘PD-1 checkpoint inhibitor’, ‘PD-L1 checkpoint inhibitor’, ‘cutaneous adverse event’, ‘clinical efficacy’, ‘clinical study’, and so on. Duplicate citations were removed.

### Eligibility and study selection

Two authors (F.M.Z. and J.J.Z.) screened the titles and abstracts of all articles obtained and assessed the full text of the selected studies in detail for eligibility. Disagreements were resolved through discussion with a third reviewer (T.Y.S.). We selected potentially eligible studies that were conducted in the real world or clinical trials, had a prospective or retrospective observational design, used PD-1/PD-L1 checkpoint inhibitors as treatment, considered self-reported or oncologist/dermatologist’s diagnosis as exposure, and considered the ORR, OS, and PFS as the primary outcomes of interest. We excluded reviews, protocols, meta-analyses, commentaries, or studies that were published only in abstract form.

### Data extraction and quality assessment

One author (F.M.Z.) extracted data from each cohort study using prespecified data extraction tables. Another author (R.Y.) checked the data extracted from each study to ensure all the data were extracted correctly. Core baseline and outcome data were extracted, including the first author, year of publication, region/country, study type, tumour type, number and age of the included participants, male sex proportion, follow-up duration, intervention, treatment time, and study outcomes. The main outcomes were ORR, HRs and 95% CIs of the OS, and HRs and 95% CIs of the PFS. We also recorded the statistical incidence rates of irCAEs, CR, PR, SD, and PD as secondary outcomes, when available. Information was obtained from published data or calculated using raw chart data. For the studies that only reported the Kaplan–Meier curve for the PFS and/or the OS, the survival data were extracted using Engauge Digitiser 12.1 and then converted into the corresponding HR and 95% CI using the EXCEL program file for calculating lnHR and SelnHR provided by Tierney et al.^49^.

The Newcastle–Ottawa Scale (NOS) was used to assess the characteristics and quality of the methods used in the included studies. This scale is based on a star system and includes three broad perspectives: selection, comparability, and exposure^50^. The total score was calculated by adding the scores for each criteria. Studies were considered good quality if the total score was at least 7/9, and studies with scores ≥ 5 were regarded as moderate-quality trials. The NOS was selected because it was validated and adaptable to our meta-analysis. Two reviewers (F.M.Z. and J.J.Z.) independently conducted quality assessments of the included studies. Any doubts were resolved through discussion and further review.

### Data synthesis and statistical analysis

We used the STATA version 15.1 software package to conduct fixed- and random-effects meta-analyses. Statistical heterogeneity was reported as the Q statistic and I^2^ statistics^51^. When *P* > 0.05 and I^2^ < 50%, it indicates that the homogeneity of a study is good, and the fixed effect model combined effect size is used. However, when *P* ≤ 0.05, or I^2^ ≥ 50%, it indicates that the homogeneity of the study was poor, and the random-effects model was used for correction. When significant heterogeneity was found between studies, we performed a subgroup analysis to examine the possible sources of heterogeneity.

We calculated the overall event rates by dividing the total number of patients across the trials with a given irCAE by the total number at risk. For analyses of binary efficacy indicators such as CR, PR, SD, PD, and ORR, we calculated the overall RR and the corresponding 95% CIs.

OS and PFS were time-to-event data, and the source of this data was obtained via longitudinal observation and follow-up. Its distribution did not follow the normal distribution. At the same time, data in these parameters were censored. Hence, we analysed the hazard ratio (HR) and 95% CI obtained directly or indirectly from the included studies.

Publication bias was assessed through a visual inspection of the funnel plots. A formal statistical assessment of funnel plot asymmetry was performed using Egger’s regression asymmetry test and Begg’s test^52^. We considered *P* < 0.05 to be evidence of small study effects. Moreover, we also conducted a sensitivity analysis. Each cohort study was excluded to examine the influence of that study on the overall estimates. The trim-and-fill method was used to detect the effect of probable missing studies on the overall effect.

### Patient and public involvement

No patients or the public were involved in setting the research question or outcome measures, nor were they involved in developing plans for the recruitment, design, or implementation of the study. No patients or the public were asked to advise on the interpretation or writing of the results. We did not directly contact individual participants in the original studies. Therefore, ethical approval was not required for this study.

## Supplementary Information

Supplementary Information.

## Data Availability

All data analysed during this study are available in the public domain.
