# Supplementary material for: Cutaneous adverse events in patients treated with PD-1/PD-L1 checkpoint inhibitors and their association with survival: a systematic review and meta-analysis
Source: Sci Rep. 2022 Nov 21;12:20038. doi: 10.1038/s41598-022-24286-3 (PMC9681870; doi:10.1038/s41598-022-24286-3)
Supplement: Supplementary file 1 — Supplementary Information. [file 41598_2022_24286_MOESM1_ESM.docx]

**Cutaneous adverse events in patients treated with PD-1/PD-L1 checkpoint inhibitors and their association with survival: A systematic review and meta-analysis**

Fangmin Zhao^1*^, Junjing Zhu^2*^, Rui Yu^1*^, Tianyu Shao^1^, Shuyi Chen^3^, Gaochenxi Zhang^3^, Qijin Shu^3^

^1^ Department of First Clinical Medical College, Zhejiang Chinese Medical University, Hangzhou 310053, Zhejiang, China

^2^ Department of Oncology, Jiaxing Hospital of Traditional Chinese Medicine Affiliated to Zhejiang Chinese Medical University, Jiaxing 314033, Zhejiang, China

^3^ Department of Oncology, The First Affiliated Hospital of Zhejiang Chinese Medical University, Hangzhou 310006, Zhejiang, China

Corresponding: Qijin Shu, Department of Oncology, The First Affiliated Hospital of Zhejiang Chinese Medical University, Hangzhou 310006, China, Email: [shuqjhz@163.com](mailto:shuqjhz@163.com)

*These authors contributed equally to this work

**Supplementary Table S1.** General characteristics of included studies.

| **Author, country** | **Study design** | **Tumor type** | **Patients** | **Male sex%** | **Age (years)** | **Median follow-up** | **Censoring** | **Intervention** | **Outcomes*** |
| --- | --- | --- | --- | --- | --- | --- | --- | --- | --- |
| Freeman-Keller2016, the United States | Retrospective | Advanced Melanoma: III-IV | 148 | Tatal: 87/58.8% | Total: NR | 138- 140week | 5(at OS analysis) | Nivolumab, dose NR, q2w | 1, 2, 3, 9 |
|  |  |  |  |  |  |  |  |  |  |
| Nakamura 2017, Japan | Retrospective | Advanced Melanoma: III-IV | 35 | Total: 18/51.4%  IrCAEs: 4/44.4%  Non-irCAEs: 14/53.8% | Total: NR  IrCAEs: 65.3(40-79)  Non-irCAEs: 68.7(41-85) | NR | 2 | Nivolumab 2 mg/kg, q3w | 1, 2, 3, 4, 5, 6, 7, 8, 9 |
|  |  |  |  |  |  |  |  |  |  |
|  |  |  |  |  |  |  |  |  |  |
| Akano2019, Japan | Retrospective | Advanced or recurrent NSCLC: IIA 1, III-IV 78 | 79 | Total: 58/73.4% | Total: 70（41-86） | NR | 0 | Nivolumab 3mg/kg，q2w | 1, 3, 10, 11 |
| Quach2019, the United States | Retrospective | Advanced Melanoma: III-IV | 318 | Total: 202/63.5%  IrCAEs: 74/61.7%  Non-irCAEs: 128/64.6% | Total: 63(22-89)  IrCAEs: 63(25-89)  Non-irCAEs: 63(22-87) | NR | 0 | Anti–PD-1(the specific name NR), dose NR, cycle NR | 1, 3, 11 |
|  |  |  |  |  |  |  |  |  |  |
|  |  |  |  |  |  |  |  |  |  |
| Lee2019, Korea | Retrospective | Multiple cancer: stages NR | 211 | Total: 137/64.9%:  IrCAEs: 21/60.0%  Non-irCAEs: 116/65.9% | Total: 61(21-91)  IrCAEs: 59.91±11.93  Non-irCAEs: 59.41±13.06 | NR | 0 | Nivolumab or Pembrolizumab, dose NR, cycle NR | 1, 2, 8 |
|  |  |  |  |  |  |  |  |  |  |
|  |  |  |  |  |  |  |  |  |  |
| Aso2020, Japan | Retrospective | Advanced NSCLC: specific stages NR | 155 | Total: 117/75.5%  IrCAEs: 42/82.4%  Non-irCAEs: 75/72.1% | Total: 68(31-88)  IrCAEs: 68(36-88),  Non-irCAEs: 69(31-88) | NR | 10(at PFS analysis), 1(at OS analysis) | Nivolumab 3 mg/kg, q2w or Pembrolizumab 200 mg, q3w | 1, 2, 3, 4, 5, 6, 7 8, 9 |
|  |  |  |  |  |  |  |  |  |  |
|  |  |  |  |  |  |  |  |  |  |
| Bottlaender 2020, France | Prospective | Advanced Melanoma: IV | 189 | Total: 109/57.7%  IrCAEs: 22/56.4%  Non-irCAEs: 87/58.0% | Total: 64.6(50.0-74.7)  IrCAEs: 66.1(50-76.4),  Non-irCAEs: 64.5(51.1–74.7) | NC | 0 | Pembrolizumab or Nivolumab, dose NR, cycle NR | 1, 2, 7, 10, 11 |
|  |  |  |  |  |  |  |  |  |  |
|  |  |  |  |  |  |  |  |  |  |
| Chan2020, Australia | Prospective | Advanced Melanoma: IIIC/IV | 82 | Total: 51/62.2% | Total: 59.9(19-82) | 40.7month | 0 | Pembrolizumab or Nivolumab at or above FDA-approved doses, cycle NR | 1, 3, 6, 7, 8 |
| Khan 2020, Europe, North America, and the Asia-Pacific region | Retrospective | Advanced or metastatic Bladder cancer: specific stages NR | 888 | Total: NR | Total: NR | NR | NR | Atezolizumab 1200 mg, q3w | 9 |

NR, not reported; NC, not clear, CR, complete response; PR, partial response; SD, stable disease; PD, progressive disease; ORR, objective response rate; PFS, progression-free survival; OS, overall survival; HR, hazard ratio; CIs, confidence intervals.

*Outcomes: irCAEs incidence (1), median time of onset of irCAEs (2), ORR (3), CR (4), PR (5), SD (6), PD (7), HR with 95%CIs of PFS (8), HR with 95%CIs of OS (9), Kaplan –Meier curve of PFS (10), Kaplan –Meier curve of OS (11).

**Supplementary Table S2.** Newcastle-Ottawa scale scores and quality assessment of included studies.

| **Study** | **Selection** | | | | **Comparability** | **Outcome** | | | **Quality*** |
| --- | --- | --- | --- | --- | --- | --- | --- | --- | --- |
|  | **Representativeness** | **Selection** | **Ascertainment** | **Outcome** |  | **Assessment** | **Follow-up** | **Adequacy** |  |
| Freeman-Keller 2016 | * | * | * | * | * | * | * | * | Good (8) |
| Nakamura 2017 | * | * | * | * | ** | * | * | * | Good (9) |
| Akano 2019 | * | * | * | * | ** | * | - | * | Good (8) |
| Quach 2019 | * | * | - | * | ** | * | - | * | Good (7) |
| Lee 2019 | * | * | * | * | ** | * | - | * | Good (8) |
| Aso 2020 | * | * | * | * | ** | * | * | * | Good (9) |
| Bottlaender 2020 | * | * | * | * | ** | * | * | * | Good (9) |
| Chan 2020 | * | * | * | * | * | * | * | * | Good (8) |
| Khan 2020 | * | * | * | - | * | * | - | - | Moderate (5) |

*Total stars awarded.

**Supplementary Table S3.** Characteristics of irCAEs in the included studies.

| **Study** | **IrCAEs occurrence** | **Diagnostic criteria** | **irCAEs type** | **irCAEs grade, No. 1-2/ ≥3** | |
| --- | --- | --- | --- | --- | --- |
| Freeman-Keller 2016 | 86/148 | NCI-CTCAE | Rash: 67  Vitiligo: 19 | | 64 / 3  19 / 0 |
| Nakamura 2017 | 9/35 | NCI-CTCAE | Vitiligo: 9 | | 8 / 1 |
|  |  |  |  |  |  |
| Akano 2019 | 14/79 | NR | Rash: 14 | | 14 / 0 |
|  |  |  |  |  |  |
| Quach 2019 | 120/318 | NCI-CTCAE | NR | | NR |
|  |  |  |  |  |  |
|  |  |  |  |  |  |
| Lee 2019 | 35/211 | NCI-CTCAE | Pruritus: 15  Eczema: 10  Maculopapular rash: 8  Dry mouth: 4  Urticaria: 4  Dry skin: 2  Alopecia: 1  Hyperpigmentation: 1  Vitiligo: 1 | | 31 / 4 |
|  |  |  |  |  |  |
|  |  |  |  |  |  |
|  |  |  |  |  |  |
|  |  |  |  |  |  |
|  |  |  |  |  |  |
|  |  |  |  |  |  |
|  |  |  |  |  |  |
|  |  |  |  |  |  |
| Aso 2020 | 51/155 | NCI-CTCAE | Pruritus: 21  Rash: 19  Erythema: 9  Other: 2 | | 21 / 0  18 / 0  6 / 3  2 / 0 |
|  |  |  |  |  |  |
|  |  |  |  |  |  |
|  |  |  |  |  |  |
| Bottlaender 2020 | 39/189 | NCI-CTCAE | rash: 18  vitiligo: 16  Pruritus: 5  Grover: 2  Sarcoidosis: 3  Vasculitis: 2  Dermatomyositis: 1  Psoriasis: 2 | | 17 / 1  16 / NR  4 / 1  2 / 0  3 / 0  1 / 1  0 / 1  2 / 0 |
|  |  |  |  |  |  |
|  |  |  |  |  |  |
|  |  |  |  |  |  |
|  |  |  |  |  |  |
|  |  |  |  |  |  |
|  |  |  |  |  |  |
|  |  |  |  |  |  |
| Chan 2020 | 33/82 | Based on clinical features and supported by histopathology when necessary | NR | | NR |
| Khan 2020 | NR/888 | NCI-CTCAE | Psoriasis: NR  Vitiligo: NR  Atopic dermatitis: NR | | NR |
|  |  |  |  |  |  |
|  |  |  |  |  |  |

NCI-CTCAE, the National Cancer Institute's Common Terminology Criteria for Adverse Events; NR, not reported.


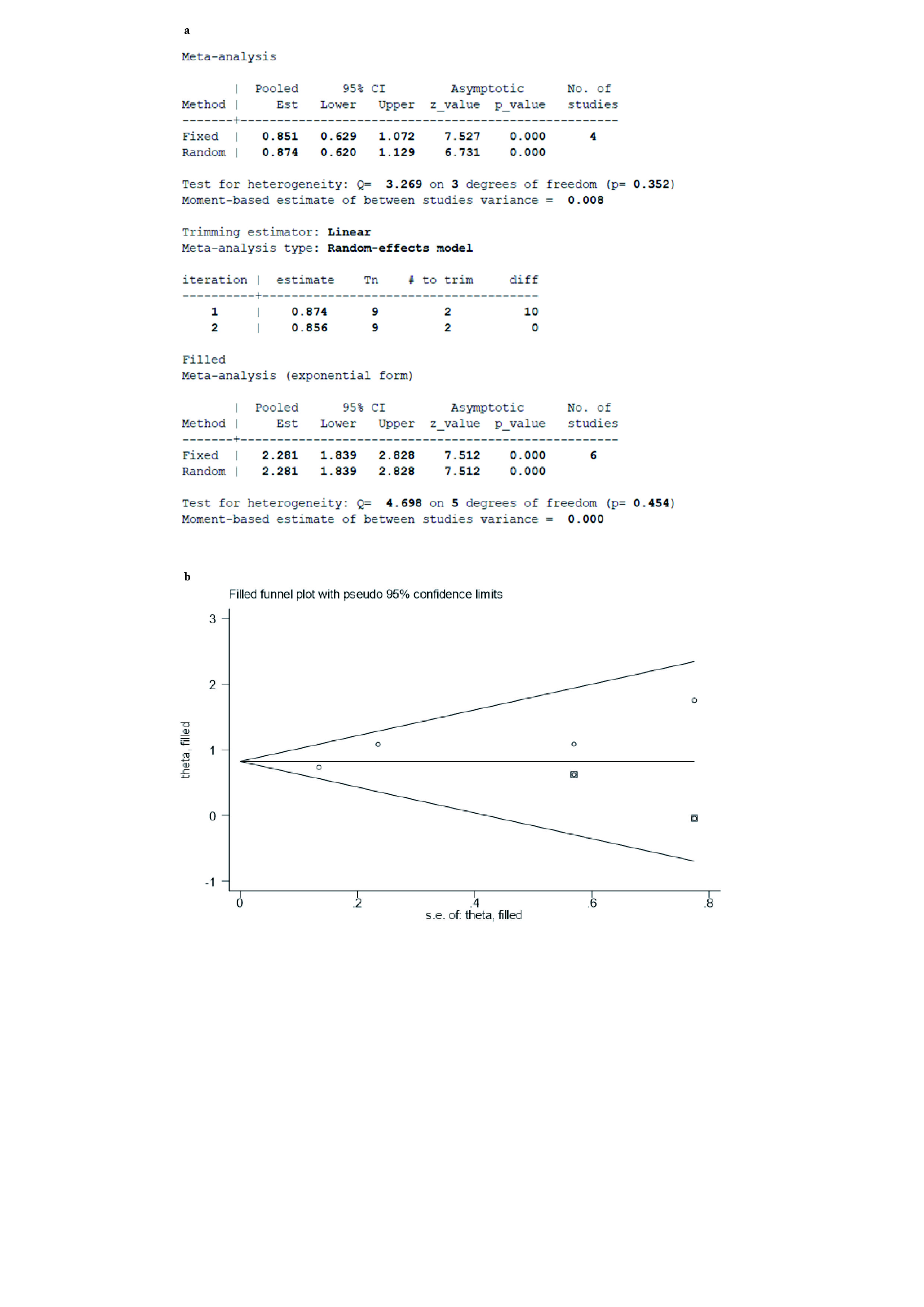


**Supplementary Figure F1.** The results of ORR’s trim and filling method. (a) Data results by trim and filling method, (b) Funnel plot after supplementing 2 studies.
